# USP7 Inhibition Alleviates H_2_O_2_-Induced Injury in Chondrocytes via Inhibiting NOX4/NLRP3 Pathway

**DOI:** 10.3389/fphar.2020.617270

**Published:** 2021-01-29

**Authors:** Gang Liu, Qingbai Liu, Bin Yan, Ziqiang Zhu, Yaozeng Xu

**Affiliations:** ^1^ Department of Orthopaedics, The First Affiliated Hospital of Soochow University, Suzhou, China; ^2^ Department of Orthopaedics, The Second Affiliated Hospital of Xuzhou Medical University, Xuzhou, China; ^3^ Department of Orthopaedics, The Affiliated Lianshui County People’s Hospital of Kangda College of Nanjing Medical University, Huai’an, China; ^4^ Department of Orthopaedics, Taixing People’s Hospital, Taixing, China

**Keywords:** osteoarthritis, ubiquitin-specific proteases, reactive oxygen species, inflammation, Pyroptosis

## Abstract

Osteoarthritis (OA), the most common form of arthritis, is a very common joint disease that often affects middle-aged to elderly people. However, current treatment options for OA are predominantly palliative. Thus, understanding its pathological process and exploring its potential therapeutic approaches are of great importance. Rat chondrocytes were isolated and exposed to hydrogen peroxide (H_2_O_2_) to mimic OA. The effects of H_2_O_2_ on ubiquitin-specific protease 7 (USP7) expression, reactive oxygen species (ROS) levels, proliferation, inflammatory cytokine release, and pyroptosis were measured. USP7 was knocked down (KD) or overexpressed to investigate the role of USP7 in OA. Co-immunoprecipitation (Co-IP) was used to study the interaction between USP7 and NAD(P)H oxidases (NOX)4 as well as NOX4 ubiquitination. NOX4 inhibitor was applied to study the involvement of NOX4 in USP7-mediated OA development. USP7 inhibitor was given to OA animals to further investigate the role of USP7 in OA *in vivo*. Moreover, H_2_O_2_ treatment significantly increased USP7 expression, enhanced ROS levels, and inhibited proliferation in rat chondrocytes. The overexpression of USP7 enhanced pyroptosis, ROS production, interleukin (IL)-1β and IL-18 levels, and the expression level of NLRP3, GSDMD-N, active caspase-1, pro-caspase-1, matrix metalloproteinases (MMP) 1, and MMP13, which was abolished by ROS inhibition. The USP7 KD protected rat chondrocytes against H_2_O_2_-induced injury. Co-IP results showed that USP7 interacted with NOX4, and USP7 KD enhanced NOX4 ubiquitinylation. The inhibition of NOX4 blocked the pro-OA effect of USP7. Moreover, the USP7 inhibitor given to OA animals suppressed OA *in vivo*. USP7 inhibited NOX4 ubiquitination for degradation which leads to elevated ROS production. ROS subsequently activates NLPR3 inflammasome, leading to enhanced production of IL-1β and IL-18, GSDMD-N-dependent pyroptosis, and extracellular matrix remodeling. Thus, UPS7 contributes to the progression of OA via NOX4/ROS/NLPR3 axis.

## Introduction

Osteoarthritis (OA), the most common form of arthritis, is a joint disease that affects older people with a lifetime risk of ∼45% (Murphy et al., 2008; Neogi, 2013). Pain and cartilage extracellular matrix (ECM) destructions are the primary symptom and features of OA (Shi et al., 2019). Variable degrees of synovial inflammation and elevated reactive oxygen species (ROS) were also found in OA (Takata et al, 2020; Lepetsos and Papavassiliou, 2016). Moreover, OA is considered a primary reason for disability in elder people (Hunter, 2011; Vos et al., 2012). However, current OA treatments are mostly palliatives, such as pain control and anti-inflammation. Therefore, understanding its pathological process and exploring its potential therapeutic approaches are of great importance.

NAD(P)H oxidases (NOXs) are ubiquitous in humans and are involved in many pathophysiologies of the diseases (Altenhofer et al., 2015). One of the members of the NOX family, NOX4, has been shown to generate ROS (Weyemi et al., 2012; Tang et al., 2018). In addition, NOX4 was recently reported to be overexpressed in OA patients, and NOX4-derived ROS production has been shown to play an essential role in the OA process (Drevet et al., 2018). Studies also suggest that NOX4 upregulation increases pyroptosis, and NOX4 inhibition attenuates pytoptosis in mice with dilated cardiomyopathy (Zeng et al., 2020). Increasing pieces of evidence show that proinflammatory cytokines participate in OA, and IL-1β and IL-18 play a crucial role in the progression of OA (Kapoor et al., 2011; Ding et al., 2017). A previous study also showed that NOX2-derived ROS production is increased in synoviocytes from OA patients and is associated with increased levels of the Nod-like receptor protein 3 (NLRP3) inflammasome (Chenevier-Gobeaux et al., 2006; Chenevier-Gobeaux et al., 2007; Clavijo-Cornejo et al., 2016).

Ubiquitin (Ub)-specific proteases (USPs), also known as deubiquitinating enzymes, remove Ub from Ub conjugates and regulates a variety of cellular processes (Ovaa et al., 2004). For example, increased expression of several USPs, including USP7, USP9X, and USP15, promoted cell proliferation (Ovaa et al., 2004). USP7 inhibition has been indicated to limit the inflammatory response in the treatment of acute or chronic inflammation (Yuan et al., 2018). Moreover, a study showed that NOX-mediated NLRP3 inflammasome activation contributes to the degradation of articular cartilage in knee OA (Clavijo-Cornejo et al., 2016). Silencing of USP22 suppressed ROS production and inflammation while inhibition of USP14 reduced the accumulation of oxidized proteins (Lee et al., 2010; Shi et al., 2016). Increased apoptosis, inflammation, MMPs expression, and defective cell proliferation frequently occur in OA. However, the roles of USP7 in OA and whether NOX-mediated NLRP3 inflammasome activation is involved in the pro-OA effect of USP7 remains largely unknown.

Moreover, hydrogen peroxide (H_2_O_2_) can induce chondrocyte apoptosis and inflammatory factor secretion (Wang et al., 2019b). This study used H_2_O_2_ as the source of the free radicals to induce chondrocytes' injury in an early-stage model of OA and USP7 was either silenced or overexpressed to study its role in the H_2_O_2_-induced OA model. The role of NOX4, ROS, and inflammation in OA was also investigated by the administration of specific inhibitors. This study found that ubiquitin–proteasome system (UPS) 7 upregulation contributes to OA via increased NOX4, elevated ROS, activated NLPR3 inflammasome, elevated interleukin (IL)-1β and IL-18, pyroptosis, and enhanced ECM remodeling.

## Materials and Methods

### Isolation of Rat Chondrocytes

Cartilage tissue was collected from knee joints of 4-week-old Sprague–Dawley rats, washed with phosphate-buffered saline (PBS), cut into pieces, and digested with collagenase II to obtain chondrocytes.

### Immunohistochemistry Assay

Rat chondrocytes were cultured on the cover slide and incubated with antibody against collagen II (ab34712, Abcam, Shanghai, China) and antibody against SOX9 antibody (ab185230, Abcam) at 4°C overnight, followed by incubation with a secondary antibody (Biyuntian, China) at room temperature for 60 min 3,3′-diaminobenzidine (DAB; Sigma-Aldrich, Shanghai, China) and hematoxylin were used for visualization. Collagen II- or SOX9-positive cells displayed brownish yellow granules in the nucleus.

### Cell Culture and Treatment

Isolated rat chondrocytes were cultured in Hyclone Dulbecco’s Modified Eagle Medium supplemented with 10% fetal bovine serum (16000-044, Gibco, Waltham, MA, United States) and 100 U/mL penicillin (Solarbio, Beijing, China). Cells were seeded in a 96-plated well (4 × 10^3^ cells/well in 100 µL of cultured medium) and cultured at 37 °C under a humidified atmosphere of 5% CO_2_ and 95% air for 12 h before the beginning of the study. Rat chondrocytes were treated with different concentrations of H_2_O_2_ (0, 50, 100, and 200 μM). In addition, rat chondrocytes were treated with 100 μM H_2_O_2_ and transduced with USP7-silencing vector or USP7 inhibitor 5 μM P22077 (Selleck, Houston, TX, United States) treatment. Moreover, rat chondrocytes were transduced with USP7 expression vector with ROS inhibitor 50 μM apocynin (Selleck) or NOX4 inhibitor 10 μM GLX351322 (Selleck) treatment.

### Proliferation Assay

The proliferation of chondrocytes was assessed using Cell Counting Kit 8 (CCK8; Dojindo, Rockville, MD, United States) following the manufacturer's protocol. Briefly, cells were seeded into 96-well plates and incubated for 24 h before treatment. Control or treated cells (90 μL) were mixed with CCK-8 reagent (10 μL) at 0, 24, 48, or 72 h. After incubating for 1 h, the optical density at 450 nm (OD 450) was measured using a reader (Bio-Rad, Philadelphia, PA, United States).

### USP7 Overexpression

The following primers were used to amplify the coding region of USP7 (NM_152,925.2): 5′-CGG​AAT​TCA​TGA​ACC​ACC​AAC​AGC​AGC​AG-3′ (*Eco*R I; forward) and 5′-CGG​GAT​CCT​CAG​TTG​TGG​ATT​TTA​ATT​GCC-3′ (*Bam*H I; reverse). The cDNA fragment encoding USP7 was inserted into pLVX-Puro (Clontech Laboratories Inc., Mountain View, CA, United States) between the cloning sites *Eco*R I and *Bam*H I. pLVX-Puro-USP7 plasmid, along with psPAX2 and pMD2G (Addgene, Watertown, MA, United States), were packaged into lentiviruses using HEK293T (ATCC) and ViaFect (Promega, Shanghai, China). Complete medium was provided 4–6 h after transfection. Lentiviruses containing supernatant was collected at 48 and 72 h after transfection and were used to infect chondrocytes. Cells transduced with lentiviruses with empty vector were used as the control (vector).

### USP7 Knock Down (KD)

The lentivirus vector system was purchased from Addgene. The designed short-hairpin RNAs (shRNAs) were synthesized and ligated into pLKO.1 plasmid (pLKO.1-shUSP7). Plasmid sequence was verified by Majorbio Bio-Pharm Tech. Inc (Shanghai, China). The shRNA-containing plasmids were packaged into the virus using the same aforementioned method above. Chondrocytes transduced with USP7-shRNA-pLKO.1 were named the shUSP7 group. Scramble shRNA vector was used as the blank control (shNC).

### Quantitative Real-Time Polymerase Chain Reaction

RNA was extracted with TRIzol Reagent (Invitrogen, Carlsbad, CA, United States) after transduction. RevertAid First Strand cDNA Synthesis kit (#K1622, Fermentas, Waltham, MA, United States) was used for first-strand cDNA synthesis. Quantification of mRNA USP7 levels was conducted using SYBR Green polymerase chain reaction (PCR) Mix (Thermo Fisher, Shanghai, China) on ABI Prism 7300 SDS System (Applied Biosystem, Foster City, CA, United States). GAPDH was used as internal control. The primer sequences used in this study were USP7-forward, 5′-GGG​CGT​GAA​GTT​CCT​GAC​C-3′; USP7-reverse, 5′-CAT​TTG​CCA​TCC​CCT​TTG​G-3′; NOX4-forward, 5′-TGC​CCA​CTT​GGT​GAA​CGC-3′; NOX4-reverse, 5′-TCA​ACA​AGC​CAC​CCG​AAA​C-3′; GAPDH-forward, 5′-GGA​GTC​TAC​TGG​CGT​CTT​CAC-3′; and GAPDH-reverse, 5′-ATG​AGC​CCT​TCC​ACG​ATG​C-3′.

### Western Blotting

Protein expression levels in supernatants and whole-cell lysates were measured as previously described (Palazón-Riquelme et al., 2018). After boiling at 93°C for 5 min, 30 µm of proteins were resolved on 10% sodium dodecyl sulfate–polyacrylamide gel electrophoresis, followed by transfer to polyvinylidene fluoride membrane. The member was incubated with primary antibodies against USP7 (ab190183), NLRP3 (ab214185), NLRP3 (ab263899), NLRP6 (ab58705), NLRC4 (ab99860), AIM2 (ab233033), GSDMD-N (ab215203), pro-caspase-1 (ab179515), active caspase-1 p20 (ab207802), NOX1 (ab55831), NOX2 (ab129068), NOX3 (ab254572), NOX4 (ab133303), NOX5 (ab198213), MMP1 (ab137332), MMP13 (ab51072; all from Abcam), ASC (orb223237), NLRP1 (orb325922; both from Biorbyt LLC, St Louis, MO, United States), β-catenin (#8480), and GAPDH (#5174; both from Cell Signaling Technology, Danvers, MA, United States) at 4°C for 12 h, and incubated with horseradish peroxidase-conjugated secondary antibody (Beyotime Biotechnology, Shanghai, China; A0208) for 1 h at 22°C after blocking with 3% bovine serum albumin (BSA) for 1 h at 22°C. Membranes were visualized with chemiluminescence reagent (Bio-Rad) with a ChemiDoc imaging machine (Bio-Rad).

### Flow Cytometric Analysis of Pyroptosis

Pyroptotic cell death was evaluated with active caspase 1 and propidium iodide (PI) staining as previously described (Wree et al., 2014). Briefly, after 48 h of treatment, approximately 5 × 10^5^ rat chondrocytes were seeded in each well of six-well plates to grow until reaching 50% confluence. Cells were then incubated with 660-YVAD-FMK (ImmunoChemistry Technologie, Bloomington, MN, United States) following the manufacturer's instructions and with 10 μL PI (Thermo Fisher) for 15 min and analyzed using Accuri C6 flow cytometer (BD Biosciences, San Jose, CA, United States).

### Flow Cytometric Analysis of ROS Levels

ROS production was detected using DCFH-DA provided in the reactive oxygen species assay kit (S0033, Beyotime Biotechnology). Briefly, after 48 h treatment, approximately 1 × 10^6^ rat chondrocytes were seeded in each well of six-well plates to grow until reaching 50% confluence. Cells were then incubated with DCFH-DA following the manufacturer's instructions for 15–60 min at 37°C. In addition, green fluorescence at 525 nm was determined using flow cytometry.

### Enzyme-Linked Immunosorbent Assay

Concentrations of IL-1β and IL-18 in cell culture medium or rat synovial fluid were measured with rat IL-1β enzyme-linked immunosorbent assay (ELISA) Kit (MultiSciences Biotech Co., Ltd., Hangzhou, China; 70-EK301B/3) and rat IL-18 ELISA Kit (Sigma-Aldrich; RAB1147) according to the supplier's protocols. Absorbance was measured at 450 nm wavelength using a PowerWave XS (Biotek, Winooski, VT, United States).

### *In vitro* Co-immunoprecipitation and Ubiquitination Assay

Co-immunoprecipitation (Co-IP) was used to measure the interaction of USP7 and NOX4. Cell lysates extracted with RIPA buffer were incubated with anti-USP7 (Abcam; ab4080), anti-NOX4 (Abcam; ab109225), or normal IgG antibody (sc-2027; Santa Cruz Biotechnology, Dallas, TX, United States) at 4°C overnight, followed by incubation with protein A/G beads (Thermo Fisher, China) at 4°C for 2 h. The immunocomplexes were washed three times with lysis buffer on a magnetic rack and then examined by immunoblotting with anti-USP7 antibody (Abcam; ab190183), anti-NOX4 antibody (Abcam; ab133303), and anti-ubiquitin (Abcam; ab7780) antibodies.

### Tissue Specimens

OA joint cartilage samples (*n* = 12) were collected from patients (65.3 ± 4.2 years; male–female, 6:6) with OA who underwent knee arthroplasty at The First Affiliated Hospital of Soochow University between June 2015 and april 2019. Human normal joint cartilage samples were collected from four patients (68.2 ± 6.8 years old; male–female, 2:2) with trauma and no history of OA or other joint diseases at The First Affiliated Hospital of Soochow University. Patients who presented with obvious joint injury or with generalized OA were excluded from the study. The present study was approved by the Ethics Committee of The First Affiliated Hospital of Soochow University. Written informed consent was obtained from all participants of this study and all investigations were performed following the Declaration of Helsinki. All patients agreed to the use of their samples in scientific research.

### Preparation of Rat OA Model

Procedures involving animals and their care were conducted following the institutional guidelines of the Department of Laboratory Animal Research facility at The First Affiliated Hospital of Soochow University. Rat OA model was set up by injection of monosodium iodoacetate (MIA 40 mg/ml in 50 μL of 0.9% NaCl solution) into the right knee joint cavities. Rats in the treatment group received P22077 for 4 weeks consecutive 2 weeks later. Hematoxylin-eosin (H and E) staining was then done to observe histological changes.

### Statistical Analyses

The data are recorded as mean ± standard error. Student’s t-test was used to compare between the analyzed groups. Moreover, one- or two-way analyses of variance were used to compare multiples groups followed by Bonferroni's multiple comparisons test. *p* values < 0.05 were defined as significant.

## Results

### H_2_O_2_ Increased USP7 Expression, Enhanced ROS Levels, and Inhibited Proliferation in Rat Chondrocytes

**FIGURE 1:**
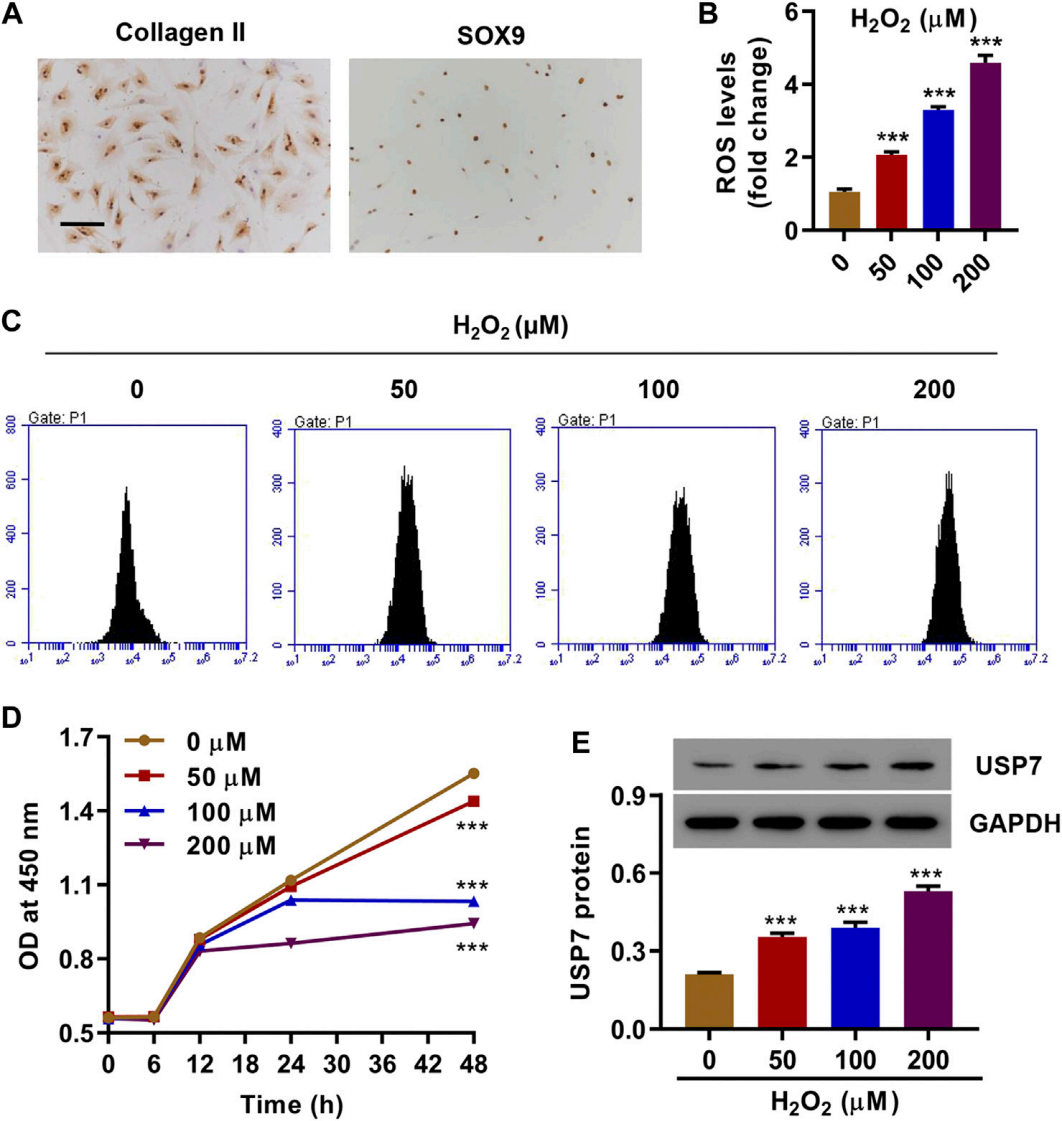
H_2_O_2_ increased USP7 expression and caused damage in rat chondrocytes. **(A)** Representative immunohistochemical staining for collagen II and SOX9 in rat chondrocytes. Rat chondrocytes were treated with different concentrations of H_2_O_2_; **(B,C)** ROS production, **(D)** cell proliferation, and **(E)** USP7 expression was measured by flow cytometry, CCK8, and Western blot, respectively. Scale bar, 100 μm ***p < 0.001 vs. 0 μM.

Chondrocytes were isolated from cartilage tissues of knee joints of rats to examine the effects of H_2_O_2_ on chondrocytes injury. Immunohistochemistry staining showed that the collected cells were collagen II- and SOX9-positive, indicating the success of chondrocyte isolation (Figure 1A). Rat chondrocytes were then treated with different concentrations of H_2_O_2_ (0, 50, 100, and 200 μM), and then ROS levels, cell proliferation, and USP7 expression were measured. Results showed that H_2_O_2_ treatment induced ROS production (Figures 1B,C), significantly inhibited proliferation (Figure 1D), and dramatically enhanced USP7 expression (Figure 1E). The involvement of USP7 in H_2_O_2_-induced proliferation inhibition and ROS production were suggested by these data.

### USP7 KD Protected Rat Chondrocytes Against H_2_O_2_-Induced Pyroptosis, ROS Production, and NLRP3 Inflammasome Activation

**FIGURE 2:**
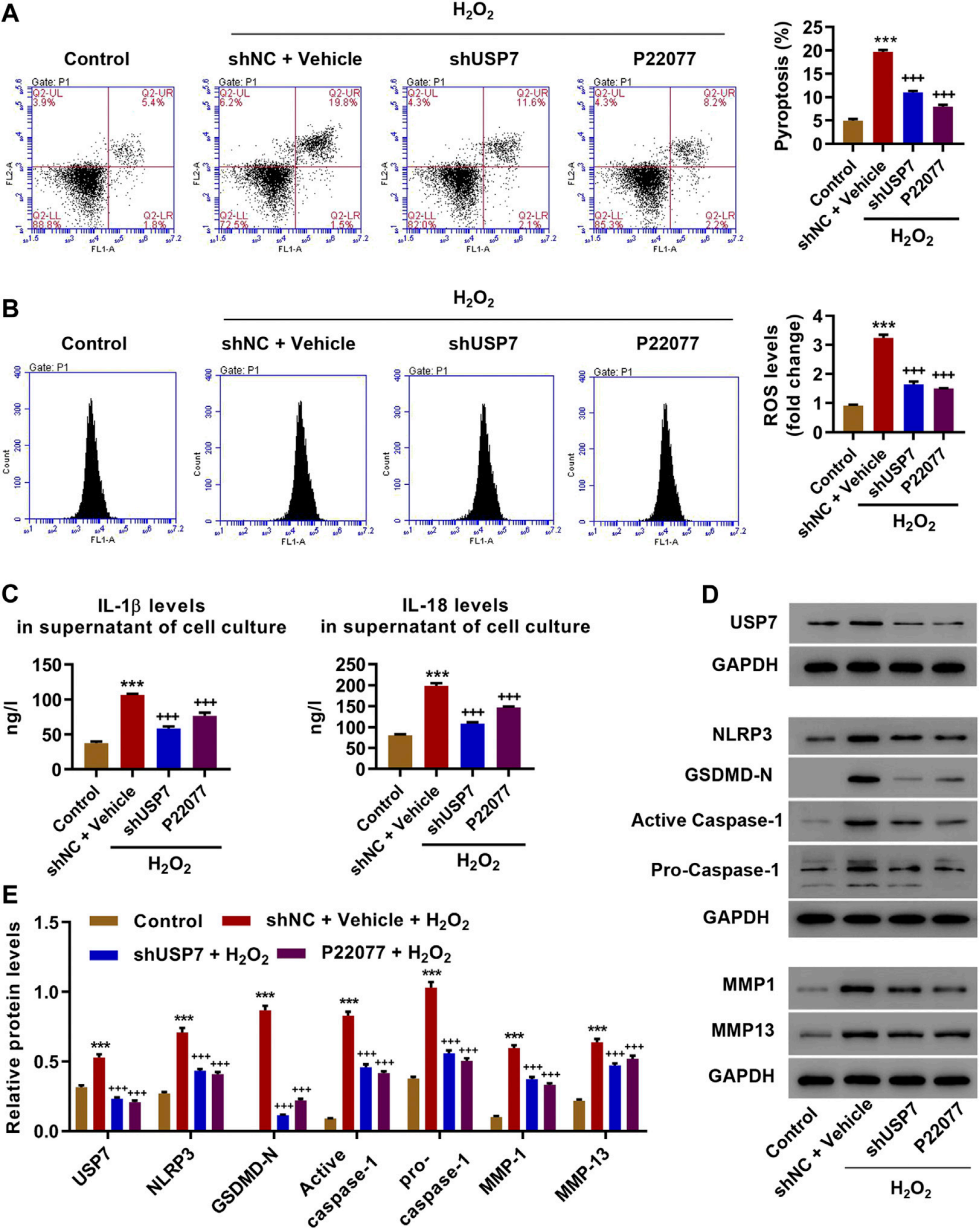
USP7 KD inhibited H_2_O_2_-induced injury in rat chondrocytes. Rat chondrocytes were treated with 100 μM H_2_O_2_ and transduced with USP7 shRNA vector or treated with USP7 inhibitor 5 μM P22077, and **(A)** pyroptosis **(B)** ROS production, **(C)** IL-1β and IL-18 levels, and **(D and E)** expression levels of USP7, NLRP3, GSDMD-N, active caspase-1, pro-caspase-1, MMP1, and MMP13 was measured by flow cytometry, ELISA, and Western blot, respectively. ***p < 0.001 vs control; ^++ +^ *p* < 0.001 vs H_2_O_2_ + shNC + vehicle.

USP7 was successfully silenced (shUSP7) compared to that of control cells (shNC; Supplementary Figures S1A,B) to study the roles of USP7 in H_2_O_2_-induced OA in chondrocytes. shUSP7- or shNC-transduced chondrocytes were treated with either USP7 inhibitor (P22077, 5 μM) or vehicle for 24 h, followed by administration of H_2_O_2_ (100 μM) for 24 h. Flow cytometry results showed that H_2_O_2_ induced pyroptosis which was blocked by either USP7 KD or USP7 inhibitor (Figure 2A). ROS assay showed that H_2_O_2_ elevated ROS production which was abolished by USP7 KD or USP7 inhibition (Figure 2B). ELISA results indicated that H_2_O_2_ treatment significantly increased IL-1β and IL-18, which was decreased by USP7 KD or USP7 inhibitor (Figure 2C). The expressions of USP7, NLRP3, GSDMD-N, active caspase-1, pro-caspase-1, MMP1, and MMP13, which was attenuated by USP7 KD or USP7 inhibitor (Figure 2D,E), were significantly enhance by the H_2_O_2_ treatment as shown in western blotting results. However, USP7 KD had no effects on the H_2_O_2_-induced expression of other inflammasomes (NLRP1, NLRP3, NLRP6, NLRC4, and AIM2) and adaptor protein ASC (Supplementary Figure S2). These data suggest that H_2_O_2_ induced ROS production, NLRP3 inflammasome activation, and pyroptosis by increasing USP7.

### USP7 Overexpression Promotes Pyroptosis and NLRP3 Inflammasome Activation in Rat Chondrocytes Through Increasing ROS Production

**FIGURE 3:**
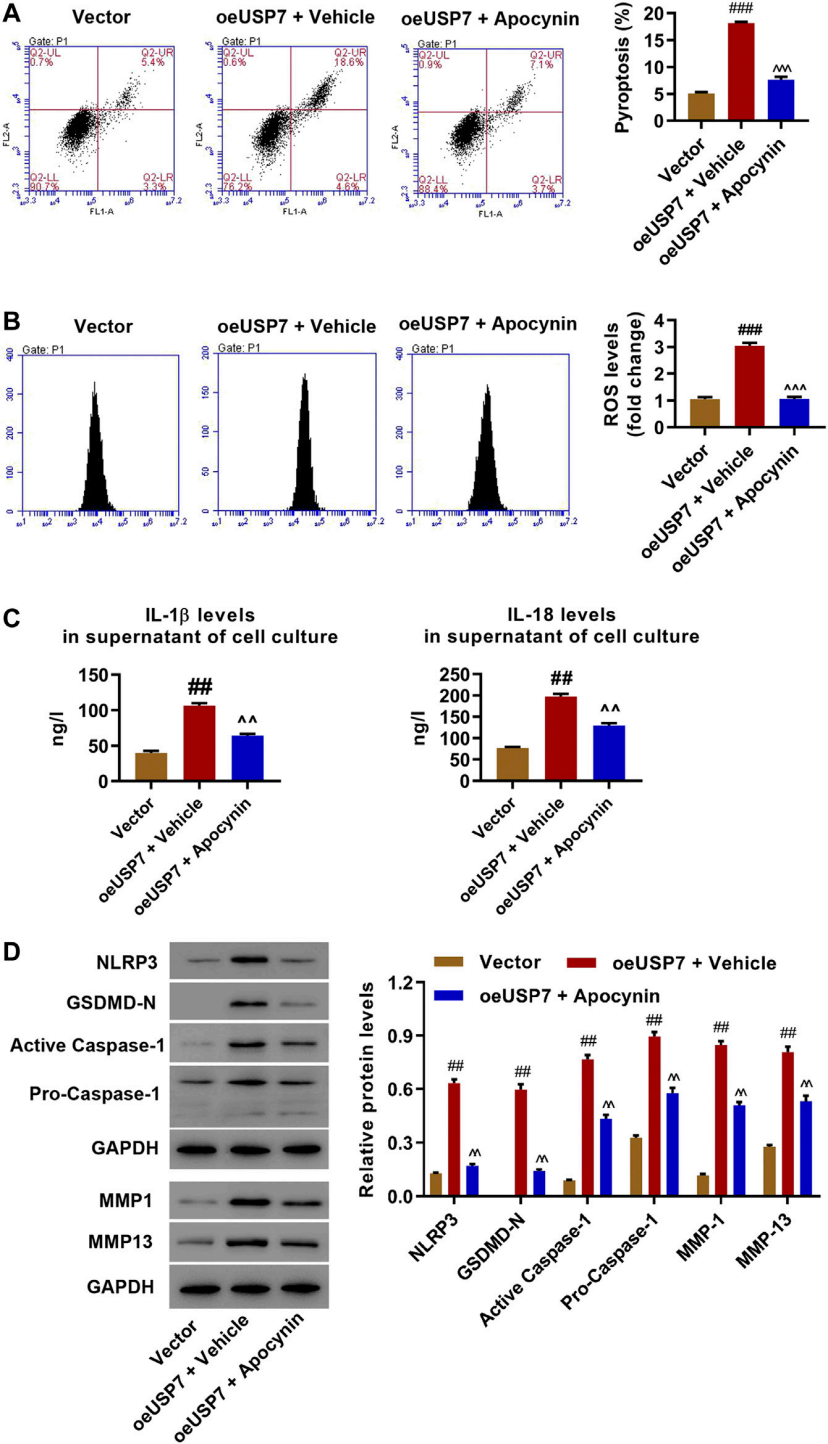
USP7 overexpression promoted injury in rat chondrocytes through increasing ROS production. Rat chondrocytes were transduced with the USP7 expression vector and treated with ROS inhibitor 50 μM apocynin, and **(A)** pyroptosis, **(B)** ROS production, **(C)** IL-1β and IL-18 levels, and **(D)** expression levels of NLRP3, GSDMD-N, active caspase-1, pro-caspase-1, MMP1, and MMP13 was measured by flow cytometry, ELISA, and Western blot, respectively. ^###^ *p* < 0.001 vs vector; ^  ^  ^*p* < 0.001 vs oeUSP7 + vehicle.

Rat chondrocytes were transduced with USP7 expression vector with or without ROS inhibitor (apocynin, 50 μM) to study the molecular mechanism by which USP7 regulates pyroptosis and NLRP3 inflammasome activation in rat chondrocytes. USP7 was successfully overexpressed (oeUSP7) compared to vector control (Supplementary Figures S1C,D). Flow cytometry results showed that USP7 overexpression induced pyroptosis and ROS production which was blocked by apocynin (Figures 3A,B). ELISA and Western blotting results indicated that USP7 overexpression significantly increased IL-1β and IL-18 levels as well as the expression of USP7, NLRP3, GSDMD-N, active caspase-1, pro-caspase-1, MMP1, and MMP13, which was decreased by USP7 KD or apocynin (Figures 3C,D). These data suggest that USP7 overexpression promotes NLRP3 inflammasome activation and pyroptosis by increasing ROS production.

### The Interaction of USP7 With NOX4 and the Enhancement of the Ubiquitinylation of NOX4 in H_2_O_2_-Induced Chondrocytes by USP7 KD

**FIGURE 4:**
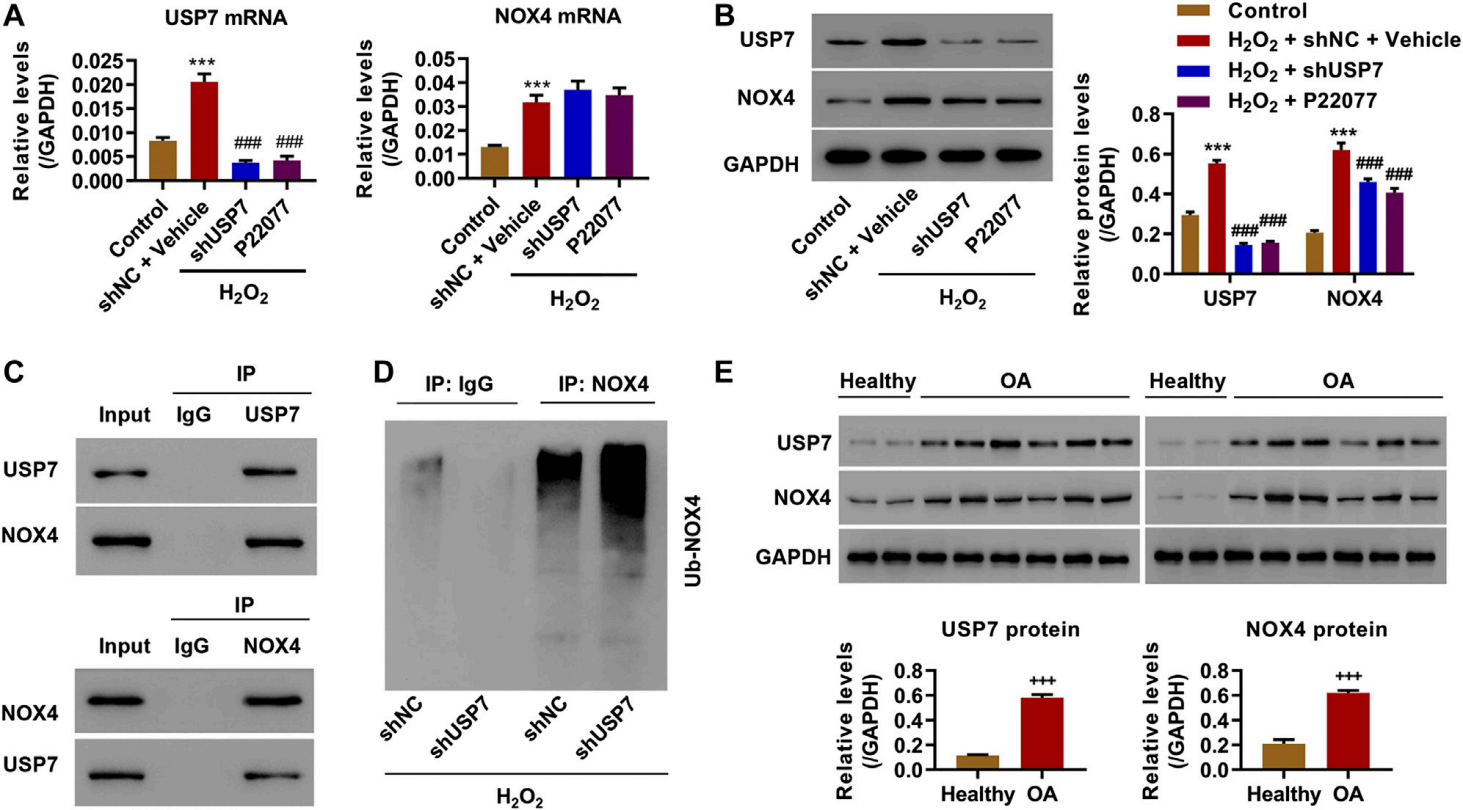
USP7 interacted with NOX4 and inhibited NOX4 ubiquitinylation. Rat chondrocytes were treated with 100 μM H_2_O_2_ and transduced with USP7 shRNA vector or treated with USP7 inhibitor P22077, and **(A)** mRNA and **(B)** protein expression levels of USP7 and NOX4 were measured by qRT-PCR and western blot. **(C)** Immunoprecipitation was carried out with IgG control, anti-USP7, or anti-NOX4 antibody. **(D)** USP7-silencing rat chondrocytes were immunoprecipitated with NOX4 or IgG antibodies and ubiquitination was evaluated by Western blot. **(E)** Expression levels of USP7 and NOX4 in the cartilage of OA patients (*n* = 12) and healthy controls (*n* = 4) were measured by qRT-PCR. ***p < 0.001 vs. control; ^###^ *p* < 0.001 vs. H_2_O_2_ + shNC + vector; ^+++^ *p* < 0.001 vs. healthy.

Previous studies showed that NOX-derived ROS production is increased in patients with OA and is associated with NLRP3 inflammasome activation (Chenevier-Gobeaux et al., 2006; Chenevier-Gobeaux et al., 2007; Clavijo-Cornejo et al., 2016). The NOXs levels were first measured after administration of USP7 inhibitor under H_2_O_2_ condition, with the lowest NOX4 protein levels, to study how USP7 affects ROS production (Supplementary Figure S3). Moreover, shUSP7 or shNC-transduced chondrocytes were treated with either USP7 inhibitor (P22077, 5 μM) or vehicle for 24 h, followed by administration of H_2_O_2_ (100 μM) for 24 h to further investigate the relationship between USP7 and NOX4 and its role in NOX4 ubiquitination. Quantitative real-time (qRT)-PCR results showed that USP7 KD or P22077 treatment significantly inhibited H_2_O_2_-induced USP7 mRNA expression. However, USP7 KD or P22077 treatment did not have a significant effect on H_2_O_2_-induced NOX4 mRNA expression (Figure 4A). Western blots showed that USP7 KD or P22077 treatment significantly inhibited H_2_O_2_-induced USP7 and NOX4 protein expressions (Figure 4B). The interaction of USP7 with NOX4 in rat chondrocytes is shown in co-immunoprecipitation (Figure 4C). Cell lysates were then immunoprecipitated with anti-NOX4 antibody and immunoblotted with anti-ubiquitin antibodies. Results showed that USP7 KD significantly increased the ubiquitination of NOX4 (Figure 4D). The expression levels of USP7 and NOX4 in cartilage from healthy people or patients with OA were then investigated. Moreover, the results indicated that patients with OA had significantly higher levels of USP7 and NOX4 compared to healthy controls. These data further confirmed the *in vitro* finding that USP7 and NOX4 were upregulated in OA, suggesting that the interaction between USP7 and NOX4 was real and not a random event.

### Inhibition of NOX4 Blocked the Effect of USP7 on Pyroptosis, ROS Production, and NLRP3 Inflammasome Activation

**FIGURE 5:**
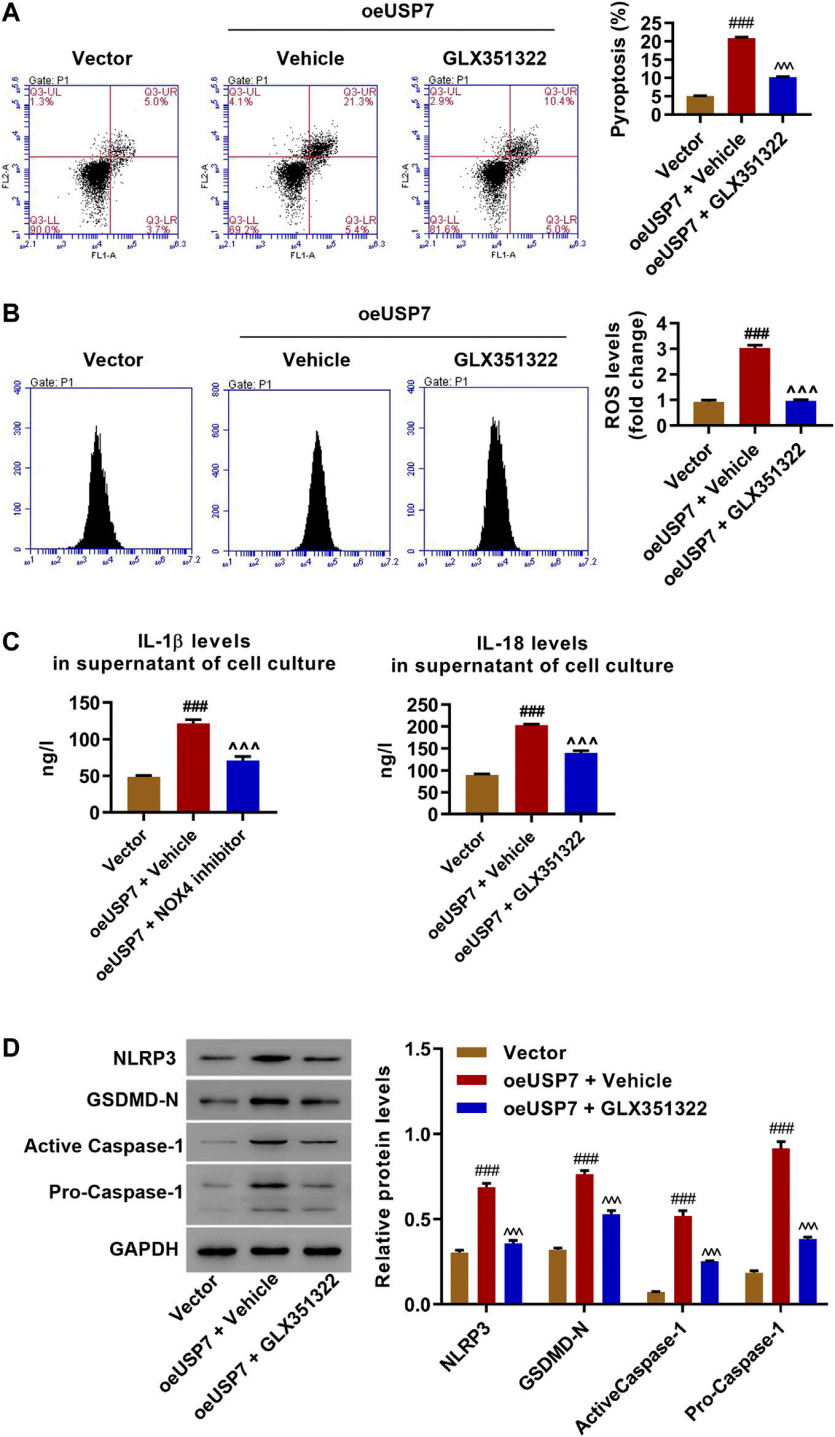
The effect of USP7 overexpression was abolished by inhibition of NOX4. Rat chondrocytes were transduced with USP7 expression vector and treated with the NOX4 inhibitor GLX351322 (10 μM), and **(A)** pyroptosis, **(B)** ROS levels, **(C)** IL-1β and IL-18 levels, and **(D)** expression levels of NLRP3, GSDMD-N, active caspase-1, and pro-caspase-1 was measured by flow cytometry, ELISA and Western blot, respectively. ^###^ *p* < 0.001 vs vector; ^  ^  ^*p* < 0.001 vs oeUSP7 + vehicle.

oeUSP7 cells and vector control cells were treated with a NOX4 inhibitor (GLX351322, 10 μM) to study NOX4’s role in USP7 regulation. Flow cytometry assay showed that inhibition of NOX4 by GLX351322 significantly decreased oeUSP7-induced pyroptosis and ROS production (Figures 5A,B). This study also found that oeUSP7-increased IL-1β and IL-18 were abolished by the inhibition of NOX4 with GLX351322 (Figure 5C). Western blotting results indicated that oeUSP7 resulted in increased expressions of NLRP3, GSDMD-N, active caspase-1, and pro-caspase-1, which was dramatically suppressed by GLX351322 (Figure 5D). These data suggest that USP7 overexpression promotes NLRP3 inflammasome activation and pyroptosis by increasing NOX4.

### Administration of USP7 Inhibitor Suppressed OA Process *In Vivo*

**FIGURE 6:**
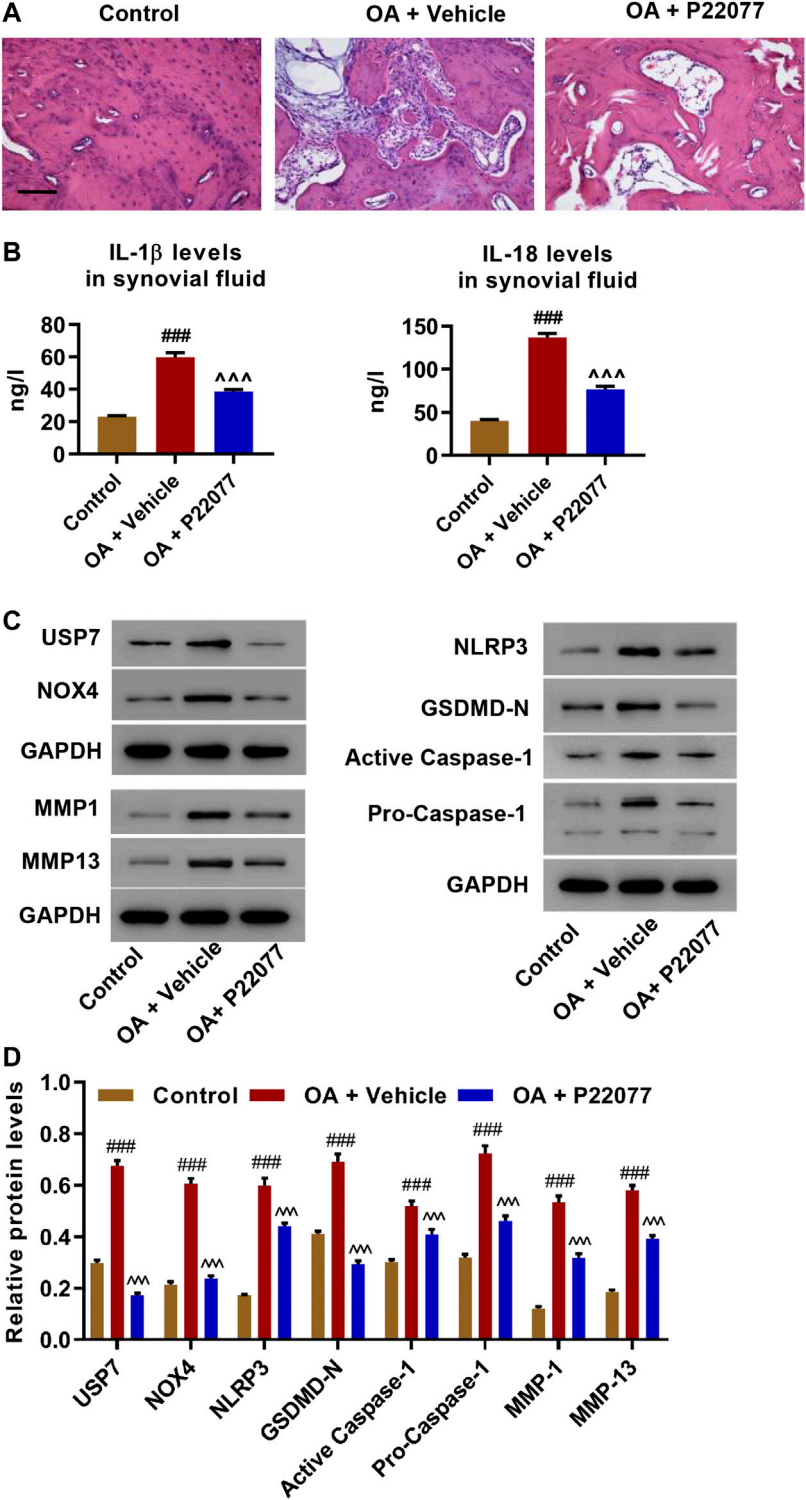
USP7 inhibition suppressed the OA process *in vivo*. Rats were injected with monosodium iodoacetate (MIA) to establish an OA model with or without P22077 treatment, and **(A)** H and E staining, **(B)** IL-1β and IL-18 levels, and **(C,D)** expression levels of USP7, NOX4, NLRP3, GSDMD-N, active caspase-1, pro-caspase-1, MMP1, and MMP13 were measured. ^###^ *p* < 0.001 vs control; ^  ^  ^*p* < 0.001 vs OA + vehicle.

MIA was used to induce OA model rats to study the role of USP7 in the OA process. Two weeks after MIA injection, USP7 inhibitor P22077 was given to rats for 4 weeks consecutive. Articular tissues were collected and H&E stained. MIA caused severe cartilage loss and the discontinuous surface of articular cartilage as shown by H&E staining. The inhibition of USP7 by P22077 significantly ameliorated MIA-induced damage (Figure 6A). Next, IL-1β and IL-18 in synovial fluid were measured. Moreover, Figure 6B shows MIA enhanced IL-1β and IL-18 which was suppressed by USP7 inhibition. Results also indicated that MIA significantly upregulated USP7, NOX4, NLRP3, GSDMD-N, active caspase-1, pro-caspase-1, MMP1, and MMP13, which was attenuated by the inhibition of USP7 with P22077 (Figures 6C,D). The involvement of USP7 in the MIA-induced OA process *in vivo* is suggested in these data.

## Discussion

OA is a degenerative disease that has a high incidence (Vos et al., 2012). However, available treatments are not enough. Thus, finding new treatment regimens and drug targets is imperative. This study used H_2_O_2_ as the source of the free radicals to induce the injury of chondrocytes in an early-stage OA model. In addition, the role of USP7 in H_2_O_2_-induced OA *in vitro* and *in vivo* was determined. Moreover, H_2_O_2_ can induce chondrocyte apoptosis and inflammatory factor secretion (Wang et al., 2019b) as well as promote inflammation by activating the NLRP3 inflammasome (Zhou et al., 2019). Thus, this study also demonstrated that H_2_O_2_ induced chondrocyte pyroptosis and proinflammatory IL-1β and IL-18 release.

Cellular proteins exist in a dynamic state with multiple degradation pathways including the ubiquitin–proteasome system (Radwan et al., 2015). Several USP family members, such as USP4, have emerged to have important roles in the OA process. For example, USP4 inhibition has been implicated in the treatment of rheumatoid arthritis (Yang et al., 2015). In addition, USP inhibition has been shown to protect mice against OA via inhibition of cytokine release and MMP13 expression (Radwan et al., 2015). Moreover, this study used H_2_O_2_ administration to chondrocytes cell to mimic OA, and another USP member, USP7, was found for upregulating in H_2_O_2_-treated rat chondrocytes and silencing of USP7 inhibited H_2_O_2_-induced pyroptosis of chondrocytes. These results indicated that USP7contributes to OA progression.

ROS are highly reactive compounds with an extremely short half-life. Moreover, they can be byproducts of numerous enzymatic reactions and can also be generated specifically by enzymes such as NADPH oxidases (Forrester et al., 2018). Numerous studies indicated that OA progression is significantly related to ROS (Henrotin et al., 2005; Li et al., 2012). Consequently, the inhibition of ROS production via NOX inhibitors have been shown effective in many models including central nervous system disorders (Barua et al., 2019), type 2 diabetes (Wang et al., 2018), and renal injury (Cha et al., 2017).

This study showed that USP7 interacted with and deubiquitinated NOX4, and the inhibition of ROS production or administration of NOX4 inhibitor significantly inhibited oeUSP7-induced pro-OA effects such as enhanced pyroptosis, elevated ROS level, increased IL-1β and IL-18 levels, and overexpression of NLRP3, GSDMD-N, active caspase-1, and pro-caspase-1. These data suggested that NOX4-dependent ROS production played a crucial role in the progression of USP7-mediated OA. Thus, USP7 regulates NLRP3 inflammasome activation, and chemical inhibition of USP7 by P22077 promotes the ubiquitination level of NLRP3 and blocks inflammasome activation (Palazón-Riquelme et al., 2018), indicating the multiple roles of USP7. Therefore, H_2_O_2_ treatment increases the protein level of NLRP3 and pro-caspase-1 due to the induction of USP7-dependent deubiquitination of NLRP3 and/or activation of NOX4/ROS/NF-κB signaling that is capable of DNA binding (Wang et al., 2019b). Moreover, P22077 is reported to target the active site of USP7 and irreversibly to inhibit its activity. However, P22077 inhibited H_2_O_2_-induced USP7 both in mRNA and protein expression. Previous studies have demonstrated that H_2_O_2_ activates NF-κB signaling in chondrocytes (Wang et al., 2019b). Moreover, USP7 deubiquitination of NF-κB subunits leads to the increase of transcriptional activity (Mitxitorena et al., 2020), suggesting this may be the mechanism by which P22077 inhibits H_2_O_2_-induced USP7 mRNA expression.

Recent studies have demonstrated that inflammasome-signaling molecules including NLRP3 are important regulators of pyroptosis and are also involved in OA progression (McAllister et al., 2018; Wang et al., 2019a). Further studies showed that ROS can activate the NLRP3 inflammasome to induce secretion of proinflammatory IL-1β and IL-18 (He et al., 2015; Chen et al., 2019). NLRP3 activation also has been shown to result in IL-1β maturation and GSDMD-dependent pyroptosis (Dubois et al., 2019). Moreover, damage of ECM underlies loss of cartilage tissue in OA (Shi et al., 2019) and the remodeling of ECM required MMPs (Ortega et al., 2004). Both of the *in vitro* and *in vivo* data in this study indicated for the first time that USP7 promoted the progression of OA via NOX4/ROS/NLPR3 signaling. The study shed light on the use of USP7 as a potential target in OA treatment.

## Conclusion

In summary, this study showed that USP7 deubiquitinates NOX4 to stabilize it, which leads to elevated ROS production. ROS subsequently activates NLPR3 inflammasome, leading to IL-1β and IL-18 elevation, GSDMD-dependent pyroptosis, and ECM remodeling. Thus, UPS7 contributes to OA progression via NOX4/ROS/NLPR3 axis.

## Data Availability Statement

The original contributions presented in the study are included in the article/Supplementary Material, further inquiries can be directed to the corresponding author.

## Ethics Statement

The studies involving human participants were reviewed and approved by The First Affiliated Hospital of Soochow University. The patients/participants provided their written informed consent to participate in this study. The animal study was reviewed and approved by The First Affiliated Hospital of Soochow University.

## Author Contributions

GL conceived the study. QL developed the methodology. BY developed the software. ZZ performed the formal analysis. YX investigated the results. GL provided the resources. YX curated the data and drafted the manuscript. GL and YX reviewed and edited the manuscript. All authors approved the final manuscript.

## Conflict of Interest

The authors declare that the research was conducted in the absence of any commercial or financial relationships that could be construed as a potential conflict of interest.
