# Supplementary material for: USP7 Inhibition Alleviates H2O2-Induced Injury in Chondrocytes via Inhibiting NOX4/NLRP3 Pathway
Source: Front Pharmacol. 2021 Jan 29;11:617270. doi: 10.3389/fphar.2020.617270 (PMC7879569; doi:10.3389/fphar.2020.617270)
Supplement: Supplementary file 1 [file table1.docx]

**USP7 inhibition alleviates** **H_2_O_2_-induced injury in chondrocytes via inhibiting NOX4/NLRP3 pathway**

Gang Liu^1,2,^*, Qingbai Liu^3,^*, Bin Yan^4,^*, Ziqiang Zhu^2^, Yaozeng Xu^1^

^1^Department of Orthopaedics, The First Affiliated Hospital of Soochow University, Suzhou 215006, China

^2^Department of Orthopaedics, The Second Affiliated Hospital of Xuzhou Medical University, Xuzhou 221000, China

^3^Department of Orthopaedics, The Affiliated Lianshui County People's Hospital of Kangda College of Nanjing Medical University, Huai'an 223400, China

^4^Department of Orthopaedics, Taixing People's Hospital, Taixing 225400, China

*Contributed equally

Corresponding author

Yaozeng Xu, Department of Orthopaedics, The First Affiliated Hospital of Soochow University, No. 899 Pinghai Road, Suzhou 215006, China. E-mail: xyzsoochow@163.com


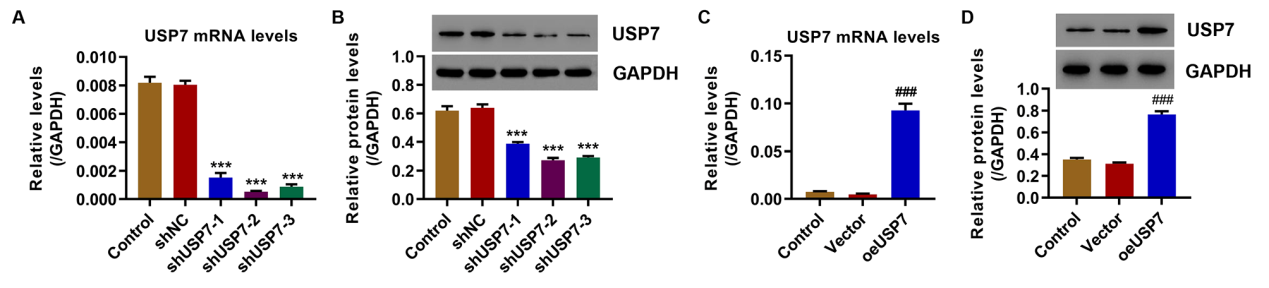


**Supplemental Figure 1.** Successful establishment of USP7 KD and USP7 overexpression in rat chondrocytes. USP7 was knocked down (**A**, **B**) or overexpressed (**C**, **D**) in rat chondrocytes using lentivirus. mRNA and proteins were measured by qRT-PCR and western blotting. *** P<0.001 vs shNC; ^###^ P<0.001 vs Vector.

**
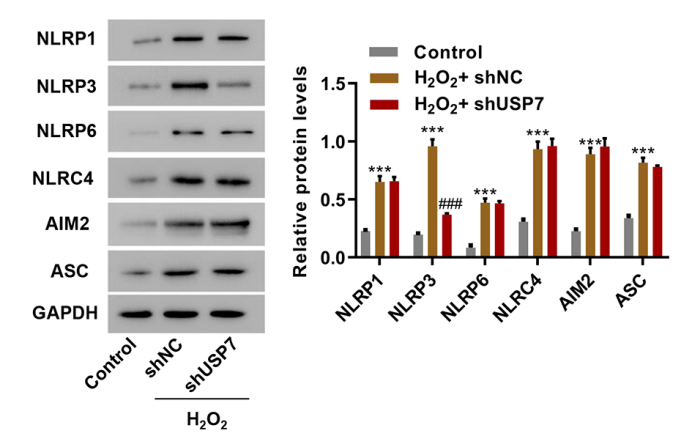
**

**Supplementary Figure 2.** Effect of USP7 KD on NLRP1, NLRP3, NLRP6, NLRC4, AIM2, and ASC expression in H_2_O_2_-stimulated rat chondrocytes. USP7 was knocked down in rat chondrocytes using lentivirus, followed by H_2_O_2_ (100 μM) treatment for 24 hours. Immunoblotting was used to check the protein levels of NLRP1, NLRP3, NLRP6, NLRC4, AIM2, and ASC. *** P < 0.001 vs. Control. ^###^ P < 0.001 vs H_2_O_2_ + shNC.

**
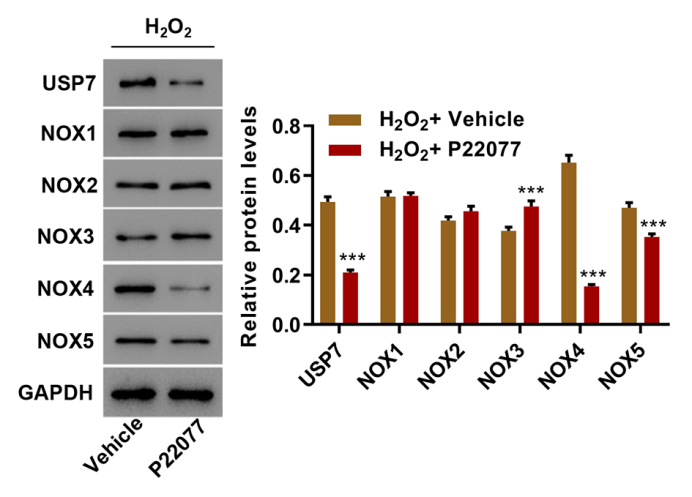
**

**Supplementary Figure 3.** Effect of USP7 inhibitor on NOX expression in H_2_O_2_-stimulated rat chondrocytes. USP7 inhibitor (P22077, 5 μM) was used to treat chondrocytes for 24 hours, followed by H_2_O_2_ (100 μM) treatment for 24 hours. Immunoblotting was used to check the protein levels of USP7, NOX1, NOX2, NOX3, NOX4, and NOX5. *** P < 0.001 vs. H_2_O_2_ + vehicle.
