# Supplementary figures and images for: USP7 Inhibition Alleviates H2O2-Induced Injury in Chondrocytes via Inhibiting NOX4/NLRP3 Pathway
Source: Front Pharmacol. 2021 Jan 29;11:617270. doi: 10.3389/fphar.2020.617270 (PMC7879569; doi:10.3389/fphar.2020.617270)

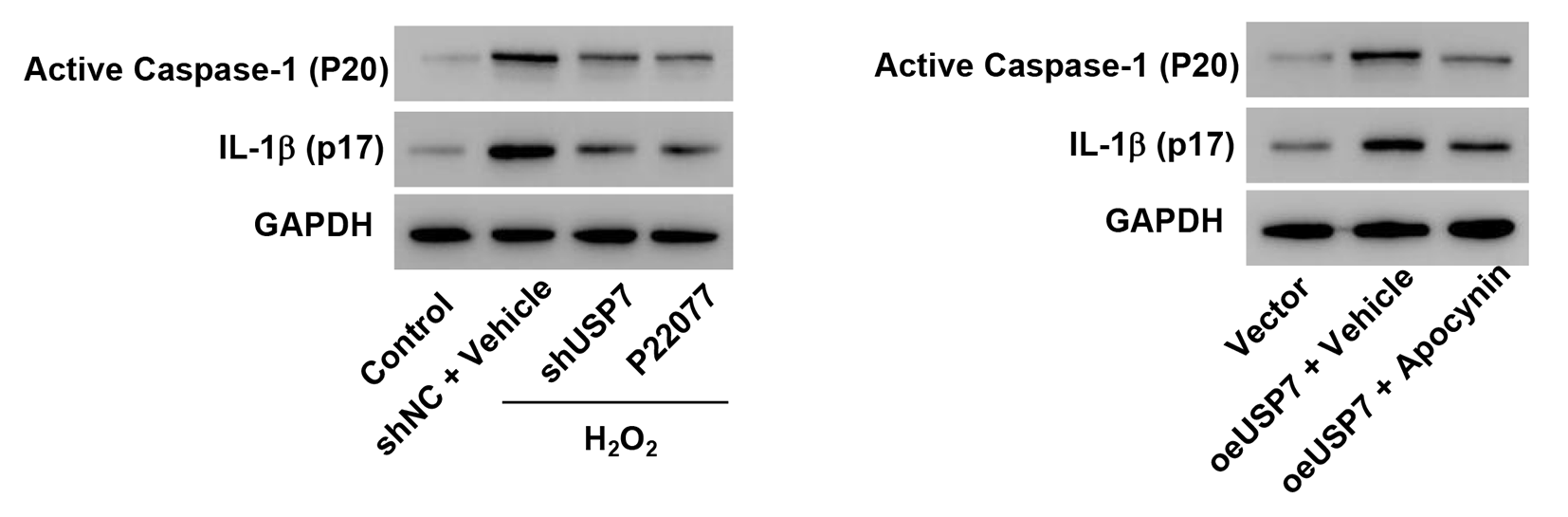

Supplement: Supplementary file 2 [file image1.tif]
